# Shift in the Distributions of Pre-existing Medical Condition, Gender and Age across Different COVID-19 Outcomes

**DOI:** 10.14336/AD.2020.1222

**Published:** 2021-04-01

**Authors:** Ming Zheng, Lun Song

**Affiliations:** Institute of Military Cognition and Brain Sciences, Academy of Military Medical Sciences, Beijing 100850, China

Dear Editor,

Recently, Dr. Nan-shan Zhong and his colleagues reported that a substantial proportion (23.7%) of coronavirus disease 2019 (COVID-19) patients in China had at least one pre-existing medical condition. The pre-existing medical conditions were identified in 21.0% of non-severe patients and 38.7% of severe patients [1]. In another study of 44,672 COVID-19 patients in China, male patients have a 1.76-fold higher fatality rate than female patients. The fatality rate also increases with patients’ age [2]. A recent article published in Aging & Disease has reported that older age and pre-existing medical conditions of hypertension and chronic obstructive pulmonary diseases were independent risk factors for the progression to severe or critical pneumonia in 564 hospitalized COVID-19 patients [3], which have raised serious concerns. However, there is scarcity of data on the distributions of the pre-existing medical conditions, gender, and age across different COVID-19 outcomes in a large cohort.

**Figure 1.**
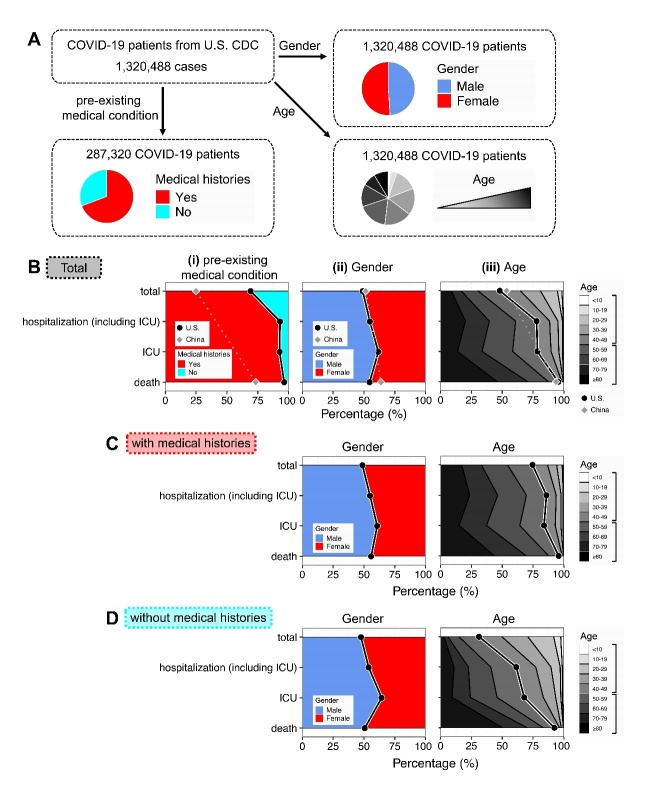
**Distributions of pre-existing medical condition, gender, and age across various disease outcomes in COVID-19 patients**. (**A**) Schematic flow diagram of COVID-19 cases from the U.S. CDC in this study. (**B**) The proportional distributions of the pre-existing medical condition, gender, and age across total COVID-19 cases and each of the subgroups with different disease outcomes; the disease outcomes were classified into the following categories: hospitalization (including ICU), ICU, and death; horizontal axes show the cumulative percentages of cases for (i) pre-existing medical condition: with and without medical histories, (ii) gender: male and female, and (iii) age: ≥ 50 years and < 50 years, with color coded according to nine split age subgroups. The black dots and solid lines represent the data from the U.S. CDC, and the grey dots and dashed lines represent the data from the Chinese CDC. (C-D) After stratification by the pre-existing medical condition, the distributions of gender and age in COVID-19 cases with (C) and without (D) medical histories.

Here, we analyzed COVID-19 surveillance data reported by the U.S. Centers for Disease Control and Prevention (CDC), from January 22nd until May 30th, 2020 [4]. Among a total of 1,320,488 confirmed COVID-19 cases, 287,320 cases had known information on pre-existing medical conditions, and 1,320,488 cases had gender and age information (Fig. 1A). Clinical outcomes of COVID-19 included hospitalization, intensive care unit (ICU) admission, and death. We found that, in the U.S. cases, the proportion of COVID-19 cases with medical histories predominated in total cases (69.2%). In addition, the proportion increased to 93.1% in hospitalized cases, 92.7% in ICU cases, and 96.4% in death cases (Fig. 1B, left plot). By contrast, in the COVID-19 surveillance data from the Chinese CDC [2], the proportion of cases with medical histories in total cases was 26.7%, which was 42.5% lower than the proportion in the U.S. cases (Fig. 1B, left plot). The difference might be due to the different definition of pre-existing medical conditions. Different from the U.S. CDC, the Chinese CDC reported cancer, while the U.S. CDC reported renal disease, liver disease, immunocompromised and neurologic disability as medical conditions. Similarly, both the U.S. and Chinese CDC reported cardiovascular diseases (including hypertension), diabetes, and chronic respiratory diseases as major medical conditions.

Next, in the U.S. COVID-19 cases, the proportion of males was 48.9% in total COVID-19 cases; by contrast, the proportions were 54.8% in hospitalized cases, 61.6% in ICU cases, and 54.5% in death cases (Fig. 1B, middle plot). Comparatively, in the Chinese cases, the proportions of males were 51.4% in total cases and 63.8% in death cases.

Next, the analysis of the age distribution revealed that, in the U.S. cases, while the proportion of older cases (≥ 50 years of age) was 48.0% in total cases, the proportions increased in subgroups of hospitalization (78.0%), ICU (78.5%), and death (95.4%). Additionally, in the Chinese cases, the proportions of older cases (≥ 50 years of age) were 53.5% in total cases and 93.7% in death cases, which showed no substantial difference to the U.S. cases. In contrast, according to the U.S. CDC data, the proportions of younger cases (< 20 years of age) were very low, ranging from 5.3% in total cases to 1.1% in hospitalized cases, 1.2% in ICU cases, and 0.1% in dead cases (Fig. 1B, right panel).

Finally, since the populations with medical histories were particularly prone to severe outcomes of COVID-19, we specifically investigated the influence of the pre-existing medical conditions on the relationships of COVID-19 outcomes with gender and age. Here, we stratified the COVID-19 cases by the pre-existing medical condition. Because the Chinese CDC did not report the stratified data, further analyses excluded the Chinese cases. We found that, among COVID-19 cases with and without medical histories, subgroups of severe outcomes tended to have higher proportions of male and older cases than the group of total cases (Fig. 1C-D).

In our study, we observed major differences in the distributions of pre-existing medical conditions, gender, and age across different COVID-19 outcomes, improving our understanding of the risk factors related to COVID-19. Among various pre-existing medical conditions in COVID-19 patients, the most frequently reported diseases were cardiovascular diseases, diabetes, and chronic respiratory diseases [1-4]. The virus that causes COVID-19 is known as severe acute respiratory syndrome coronavirus 2 (SARS-CoV-2) [5]. In order to enter the host cells, the SARS-CoV-2 spike protein binds to angiotensin-converting enzyme 2 (ACE2) on human cells [6]. It is worth noting that ACE2 is involved in the development of heart failure, hypertension, and diabetes [7, 8]. Moreover, cardiovascular diseases and diabetes are often treated with drugs that increase ACE2 expression, which might facilitate the SARS-CoV-2 infection and lead to serious and fatal consequences of COVID-19 [9]. Next, it’s reported that males have lower basal levels of immunoglobulin and lower antibody responses to viruses than females [10]. Additionally, aging immunity may exacerbate COVID-19 through increased baseline inflammation in older individuals [11]. Further studies should explore the relationships between the biological features of SARS-CoV-2, gender and age differences of immunity, and the epidemiological findings of COVID-19.

Taken together, our study indicated the importance for the COVID-19-related risk to be assessed according to pre-existing medical conditions, gender, and age subgroups. Additionally, the public health policy of COVID-19 should be made specifically for populations with medical histories of cardiovascular diseases, diabetes, and chronic lung diseases, male gender, and aged more than 50 years old.
